# A Multi-Label Classifier for Predicting the Most Appropriate Instrumental Method for the Analysis of Contaminants of Emerging Concern

**DOI:** 10.3390/metabo12030199

**Published:** 2022-02-23

**Authors:** Nikiforos Alygizakis, Vasileios Konstantakos, Grigoris Bouziotopoulos, Evangelos Kormentzas, Jaroslav Slobodnik, Nikolaos S. Thomaidis

**Affiliations:** 1 Laboratory of Analytical Chemistry, Department of Chemistry, National and Kapodistrian University of Athens, Panepistimiopolis Zografou, 15771 Athens, Greece; 2 Environmental Institute, Okružná 784/42, 97241 Kos, Slovakia; slobodnik@ei.sk; 3 National Centre for Scientific Research “Demokritos”, Institute of Informatics and Telecommunications, 15341 Agia Paraskevi, Greece; vkonstantakos@iit.demokritos.gr; 4 Department of Informatics, National and Kapodistrian University of Athens, Panepistimiopolis Zografou, 15771 Athens, Greece; greg.bouzios@gmail.com; 5 Cognity S.A., Leof. Kifisias 42, 15125 Marousi Athens, Greece; ekormentzas@cognity.gr

**Keywords:** gas chromatography, liquid chromatography, retrospective suspect screening, contaminants of emerging contaminants

## Abstract

Liquid chromatography-high resolution mass spectrometry (LC-HRMS) and gas chromatography-high resolution mass spectrometry (GC-HRMS) have revolutionized analytical chemistry among many other disciplines. These advanced instrumentations allow to theoretically capture the whole chemical universe that is contained in samples, giving unimaginable opportunities to the scientific community. Laboratories equipped with these instruments produce a lot of data daily that can be digitally archived. Digital storage of data opens up the opportunity for retrospective suspect screening investigations for the occurrence of chemicals in the stored chromatograms. The first step of this approach involves the prediction of which data is more appropriate to be searched. In this study, we built an optimized multi-label classifier for predicting the most appropriate instrumental method (LC-HRMS or GC-HRMS or both) for the analysis of chemicals in digital specimens. The approach involved the generation of a baseline model based on the knowledge that an expert would use and the generation of an optimized machine learning model. A multi-step feature selection approach, a model selection strategy, and optimization of the classifier’s hyperparameters led to a model with accuracy that outperformed the baseline implementation. The models were used to predict the most appropriate instrumental technique for new substances. The scripts are available at GitHub and the dataset at Zenodo.

## 1. Introduction

Analytical environmental chemistry focuses on the occurrence of chemicals (also known as emerging contaminants) in environmental samples [1] and the development of new analytical methods for their determination [2,3]. Traditional analytical methods focus on a list of preselected contaminants. This trend changed during the last decade after the introduction of high-resolution mass spectrometry (HRMS) detectors [4]. The combination of a separation technique, such as liquid chromatography (LC) or gas chromatography (GC) with HRMS, forms powerful analytical instrumentation, which alleviates the constraint for focusing on a limited number of chemicals [5]. The term “LC” includes many techniques (reversed-phase, HILIC, ion-exchange chromatography). However, reverse-phase liquid chromatography is the most frequently used LC separation technique for the analysis of semi-polar and polar contaminants of emerging concern. Therefore, the analysis of a sample by reversed-phase LC-HRMS (from now on simply referred to as LC-HRMS) and by GC-HRMS theoretically allows the detection of a very wide chemical universe that is contained in a given sample given analytical limitations (e.g., detection limits, sensitivity, matrix interferences, etc.).

The chemical signals that are stored in the HRMS data remain mostly underexploited because of the current limitations in software and mass spectrometric libraries [6]. To avoid the discard of overlooked chemicals, the HRMS data is stored in repositories [7]. The reason for storing this information is the possibility to return to it and search for the occurrence of any suspected contaminant to the digitally stored sample through a strategy of retrospective suspect screening [8,9]. 

Chemical regulators and policymakers come frequently with requests to scientific associations, such as the NORMAN association for retrospective suspect screening of chemicals that are potentially persistent, bioaccumulative and toxic. The NORMAN association is a self-funded independent network of reference laboratories, research centers and related organizations focused on monitoring emerging environmental substances [10]. The NORMAN association has established a novel database system (NORMAN Database System [11]) to facilitate such requests and support its research-to-policy aim [12]. The first step of suspect screening involves the decision of which type of data must be searched, LC-HRMS or GC-HRMS. 

Currently, there is a lack of prediction models addressing this knowledge gap in the field of exposomics and other omics’ disciplines. Recently, a publication focused on the construction of models to capture the physicochemical properties of molecules that determine their amenability to detection in different electrospray modes (ESI) of detection (LC positive ESI-HRMS versus LC-negative ESI-HRMS) [13]. To the authors’ knowledge, this is the first publication that attempts to predict compound amenability in different instrumentation (reversed-phase LC-HRMS versus GC–HRMS). The main reason for this lack is the absence of appropriate and large datasets to be modeled. The objective of our study was (i) to create a training and test set based on knowledge gained by the experts of NORMAN, (ii) to build a model with the highest accuracy and lowest possible complexity and (iii) apply the model to chemicals of the NORMAN Substance database to predict the type of data (LC-HRMS or GC-HRMS or both) to be investigated.

## 2. Results

### 2.1. Comparison between Feature Sets

A significant part of the modeling workflow was the feature selection (FS). FS was conducted with the aim of selecting a few of the most informative descriptors out of the 1446 descriptors. To verify the efficiency of our FS strategy, we compared the performance of the selected classifier trained on the different feature sets. We performed this evaluation under a 10 × 10-CV scheme (i.e., 10 repetitions of 10-fold CV). The results of this evaluation are shown in Table 1. Each row shows the mean accuracy and standard deviation of the evaluated classifier for the corresponding 10-fold CV. We observe that our FS was successful in greatly reducing the number of features from 1446 to 8 without sacrificing performance. To confirm our findings, we applied the Friedman test to assess if the differences in the classifiers’ performances are statistically significant. The test showed statistically significant differences (Q = 331.69, *p* < 0.0001) and rejected the null hypothesis that the classifiers being compared are alike. An effect size estimate was also calculated with Kendall’s coefficient W = 0.558 [14].

Since the result of the Friedman test was statistically significant, post hoc analysis using the Nemeyi test was done to identify the classifiers-feature sets that actually differ. The resulting *p*-values can be seen in Table 2. We observe that the final feature set is significantly different from the previous steps of our FS, but not from the initial set. Thus, the significance of the Friedman test was mainly due to the performance differences of our selected features, indicating a successful feature selection.

### 2.2. Comparison with Baseline

**Figure 1:**
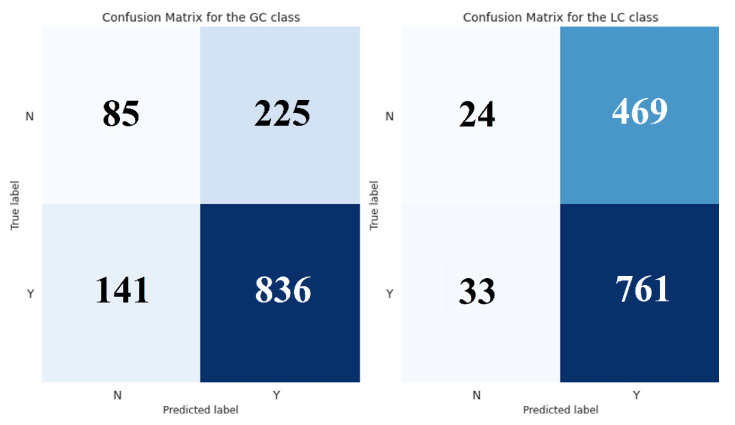
Confusion matrix—rule-based classifier. “Y” stands for “Yes”, indicating that a compound is amenable, while “N” stands for “No”, indicating that a compound is not amenable.

**Figure 2:**
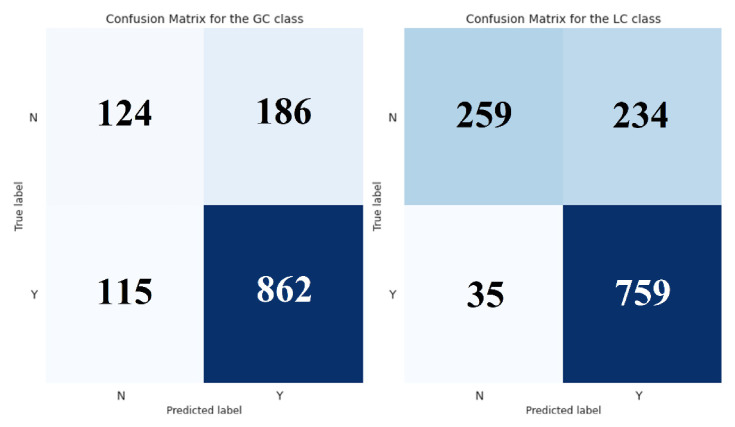
Confusion matrix—decision tree classifier. “Y” stands for “Yes”, indicating that a compound is amenable, while “N” stands for “No”, indicating that a compound is not amenable.

To validate the performance of the proposed classifier, we compared it with the implemented baseline on a holdout set. In particular, we trained a decision tree classifier (DTC) of depth 4 using the 8 selected features and obtained its predictions on the independent dataset (Figure S1). Likewise, we used the rule-based classifier (RBC) to get the predictions in the same instances. The results of this evaluation are shown in Table 3 and Table 4 and Figure 1 and Figure 2.

## 3. Discussion

### 3.1. Feature Selection (FS)

The FS strategy drastically reduced the number of descriptors from 1446 to 8 without sacrificing performance, as shown by the application of the Friedman test and the post hoc analysis using the Nemeyi test. The latter test was used to identify the classifiers-feature sets that actually differ. To further illustrate the efficiency of the FS, we compared the accuracy between the initial and final features using Wilcoxon’s signed rank test for paired scores. In particular, we performed a one-sided Wilcoxon’s test to demonstrate the superior performance of the selected features based on all the measurements (100) that were included in Table 1. That is, the null hypothesis of the test was that the classifiers’ performance does not differ significantly, while the alternative hypothesis was that the manually selected features had greater performance than all the initial ones. The Wilcoxon’s test yielded statistically significant results (W = 2888, *p* = 0.033), showing that the performance of the final features was significantly greater than the performance of all features. We also calculated relevant effect size estimates for Wilcoxon’s signed rank test [14]. Specifically, we computed the matched pairs rank biserial correlation (MPRBC), which is the difference between the proportion of favorable and unfavorable evidence; in the case of the Wilcoxon’s signed rank test, the evidence consists of rank sums [15]. In addition, we calculated the common language effect size (CLES), which is the probability that a score sampled at random from one distribution will be greater than a score sampled from some other distribution [16]. The resulting values were MPRBC = 0.215 and CLES = 0.554, indicating a probably small effect size. These findings showed that the feature selection strategy was successful. It substantially reduced the number of features from 1446 to 8, while keeping an equivalent—if not better—performance.

### 3.2. Superiority of the Decision Tree Model against the Baseline Rule-Based Classification

**Figure 3:**
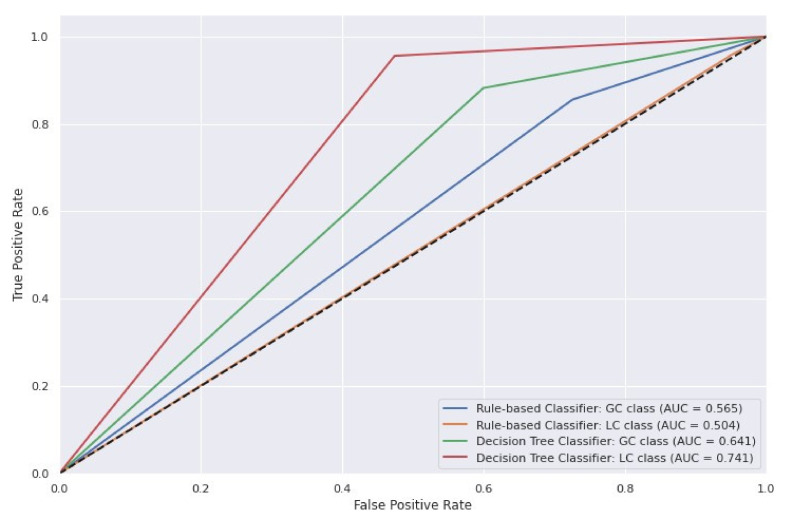
Receiver operating characteristic (ROC) curve analysis.

The performance of the proposed classifier was compared with the implemented baseline using a holdout set. Overall, the decision tree outperformed the rule-based classifier across all chosen metrics. The improved predictive power was especially evident in the LC’s classification. DTC showed a more balanced performance and greatly reduced the false-positive rates (Figure 1 and Figure 2). To further demonstrate the accuracy of our model, we performed a receiver operating characteristic (ROC) curve analysis for both classifiers (Figure 3). As we already observed, the DTC was superior to RBC on both classification tasks. The performance difference was especially evident when evaluated in the LC class, with an area under the curve value of 0.741 and 0.504, respectively.

Finally, we calculated the McNemar’s test to confirm that the observed differences were statistically significant. McNemar’s is a non-parametric test that is generally applied to compare the classification errors of two classifiers. We chose this test and not the parametric t-test, because of the difficulty of ascertaining the normality, equal variance, and sample randomness assumptions. It was also the most appropriate for our case since it is ideally applied over an independent test set for performance assessment. Besides, McNemar’s test has been successfully applied for classifiers’ comparison in previous studies [17]. Therefore, we generated the 2 × 2 contingency matrix for each class and computed the corresponding McNemar’s test. Both tests showed statistically significant results, which confirmed the superiority of our classifier. Specifically, the McNemar’s test yielded χ^2^ = 13.26, *p* = 0.0003 for the GC class, and χ^2^ = 159.72, *p* < 0.0001 for the LC class, further validating our previous observations. For completeness, we calculated the odds ratio (OR) for each class, since they can serve as effect size estimates for data subjected to McNemar’s test [18]. As expected, the OR for the GC case was 1.53, while for the LC case was 5.48, which corresponds to a small and large effect size, respectively [18]. These results demonstrated that our model performed significantly better than the baseline classifier and provided a major improvement in the task of GC-LC substance classification.

### 3.3. Application of the Decision Tree Model

**Figure 4:**
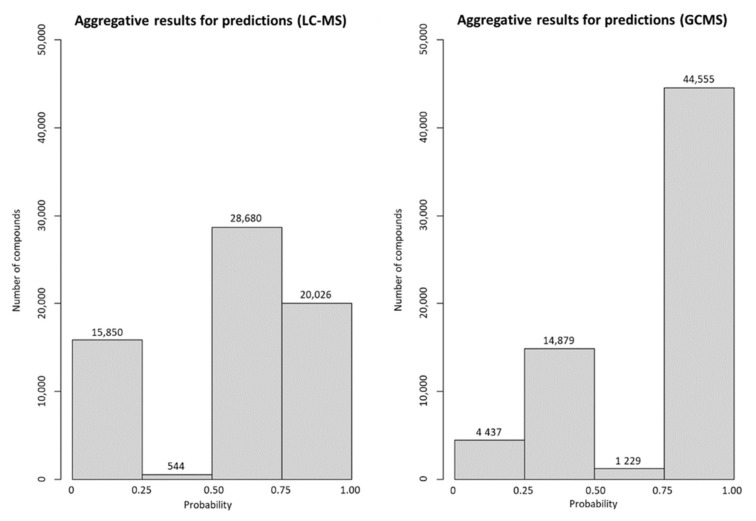
Aggregative results of LC-MS and GC-MS amenability.

The generated model was used to predict the most suitable instrumentation for 65,691 compounds included in the NORMAN SusDat as of 10th of April 2021 [19]. Predictions could be achieved for 65,100 compounds (Table S1, Figure 4). The prediction for 591 compounds was not feasible because the derivation of the descriptors was not successful mainly due to time limitation in the generation of descriptors for complex molecules (maximum time for the generation of descriptors was set to 3 min). Based on the results, 45,784 compounds were predicted to be LC-MS amenable, whereas 48,706 compounds were predicted to be GC-MS amenable. It is worth mentioning that more than 45% of the compounds were predicted to be detectable by both analytical platforms. However, the results indicate that the two analytical techniques cover unique chemical space and, in that sense, are complementary.

The predictions will be exploited for future retrospective suspect screening efforts of contaminants of emerging concern in digitally archived environmental samples [20]. 

## 4. Materials and Methods

### 4.1. Data Collection and Processing

The NORMAN Suspect List Exchange was used for the generation of the dataset [19]. Datasets with a clear label (LC or GC) were used. More specifically, we used S3 NORMANCT15, which contains a list of compounds that were detected in surface water from the Danube River in a pan-European collaborative trial employing both GC-HRMS and LC-HRMS [21]. Moreover, the GC and LC target lists were used by the following two institutes: the National and Kapodistrian University of Athens (NKUA) and the Helmholtz Centre for Environmental Research (UFZ). S21 UATHTARGETS is the LC target list of NKUA [22], S65 UATHTARGETSGC is the GC target list of NKUA and S53 UFZWANATARG contains the LC [23] and GC target lists of UFZ. Finally, two GC target lists (S51 WRIGCHRMS and S70 EISUSGCEIMS) were used. These lists contain GC substance lists and were provided by two Slovak institutes, the Water Research Institute (WRI) and the Environmental Institute. The aforementioned compound lists were merged together to form a labeled dataset. The SMILES were used to calculate 1446 molecular descriptors. An amount of 1446 descriptors were produced by the PaDEL-descriptor v 2.21 (Queenstown, Singapore) [24], logP was produced by JRgui v1.0 (North Chicago , IL, USA) [25] and the boiling point by USEPA ECOSAR v1.43 (New York, NY, USA) [26]. The dataset consisted of 6431 instances and was split into the training set (5144 instances) and test set (1287 instances; 20% of the dataset). Modeling was conducted in python 3.8.5 using sklearn. The script is available at github (https://github.com/nalygizakis/LCvsGC, accessed on 22 February 2022) and requires the following packages to run: pandas (v1.1.5), numpy (1.19.5), mlxtend (v0.14.0), matplotlib (v3.2.2), seaborn (v0.11.1), graphviz (v0.10.1), joblib (v1.0.1), pingouin (v0.3.11), scikit_learn (v0.24.2), scikit_posthocs (v0.6.7). The dataset was split into a training and test set at the beginning of the workflow before feature selection or any other operation using the function *train_test_split* of sklearn (module model_selection). The test set was held back from the training of the models and was solely used for an unbiased evaluation of the prediction skills of the models.

### 4.2. Baseline Implementation

A rule-based classifier (RBC) was implemented to serve as the baseline performance on the chosen classification task. Its rules were derived from previous knowledge of the specific domain and capture the process a person would follow to determine the nature of a substance. In particular, we used the boiling point, the molecular weight, and the polarity to classify a substance as GC (boiling point from 100 to 350 °C, molecular weight below 700 Da, logP higher than 2) while using only the polarity (logP less than 5.91) for the case of LC. Detailed information about the implementation is included in the provided source code and its documentation. 

### 4.3. Model Construction

To construct the final model for our task, we combined an algorithm comparison and a feature selection strategy. First, we defined our task as a multi-label classification problem, since an instance can belong both to the GC and LC classes. We then evaluated and selected the appropriate algorithm for our use-case. Given the nature of the problem, we chose the model with the highest performance and lowest complexity. Therefore, the decision tree classifier (DTC)—which was inferior only to the random forest classifier (RFC)—was the model of our choice.

Having selected the appropriate algorithm, we performed multiple steps of feature selection (FS) to arrive at a minimal and relevant-only feature subset. More specifically, the following FS methods were used:

#### 4.3.1. Filter Methods

The goal of filtering was to initially remove quasi-constant features. As the name suggests, these are features that are almost constant, as their values are the same for a very large subset of the observations. The variance threshold for quasi-constant filtering was set to 99%. Therefore, features that have more than 99% similar values in the observations were removed.

After the quasi-constant filtering, correlation filtering was applied. An amount of ≥2 features are correlated if they are close to each other in the linear space. Correlation between the output observations and the input features is very important and such features should be retained. However, if two or more than two features are mutually correlated, they convey redundant information to the model and hence only one of the correlated features should be retained to reduce the number of features. Since the data did not come from a specific distribution, Spearman’s rank correlation coefficient (r_s_) was chosen, which is a non-parametric correlation measure and is appropriate for both continuous and discrete ordinal variables [27]. The threshold was set to 0.9. As a result, features with their r_s_ value close to 1 were eliminated.

#### 4.3.2. Random Forest Feature Importance (RFFI)

Importance weights were calculated by training a random forest model on the filtered dataset. Features whose importance was greater than or equal to the mean value multiplied by a scaling factor of 1.5 were kept, while the others were discarded.

#### 4.3.3. Recursive Feature Elimination Methods

**Figure 5:**
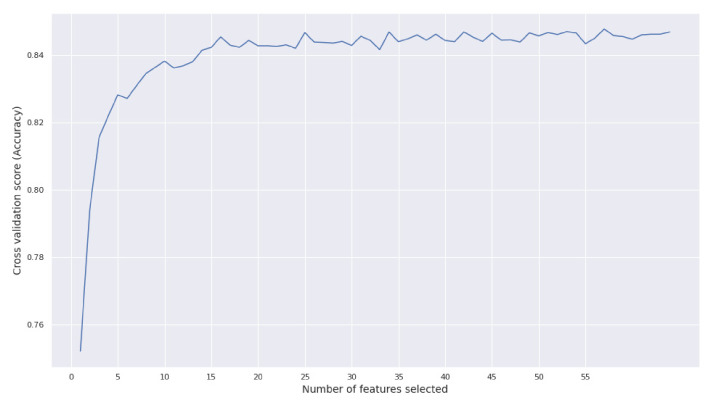
RFECV score vs. number of features.

Feature ranking with recursive feature elimination and cross-validated selection using 10 folds was achieved by training a random forest classifier. The Hamming score was used as an evaluation measure for keeping the best features. The resulting scores with the corresponding number of features can be seen in Figure 5. We noticed that the performance plateaued after the first 10 features. For this reason, our goal for the next steps of the FS was to keep the 10 (or less) top-performing features in order to gain interpretability without sacrificing accuracy.

#### 4.3.4. Sequential Feature Selection Methods

Using a random forest classifier and the features from the RFFI, the more expensive sequential forward feature selection was used. The process was repeated 5 times using a cross-validated selection of 10 folds and only keeping the best 10 features per iteration.

#### 4.3.5. Manual Feature Selection

Using the 10 selected best features, manual feature selection was performed by calculating the overlap among the 5 repetitions. The features with the best overlap, which were selected for training, are as follows:

- Minimum E-State—gmin;
- Topological polar surface area—TopoPSA;
- Boiling Point—BoilingPoint;
- nhigh lowest partial charge weighted BCUTS—BCUTc-1l;
- Number of nitrogen atoms—nN;
- Number of basic groups—nBase;
- Overall or summation solute hydrogen bond acidity—MLFER_A;
- Maximum H E-State—hmax.

Finally, the hyperparameter tuning of the model was accomplished through grid search using a cross-validated selection of 10 folds and the Hamming score for evaluation. The parameter grid is outlined below:

- criterion: [gini, entropy];
- max_features: [auto, sqrt, None];
- min_samples_split: [2, 3, 5, 8, 10, 20, 40];
- max_depth: range (3, 30);
- min_samples_leaf: [1–5, 10, 20, 40].

The best score achieved through grid search was 81.3%. The best estimator was a decision tree classifier with a max depth equal to 26, criterion “gini” and max features equal to “auto”.

**Figure 6:**
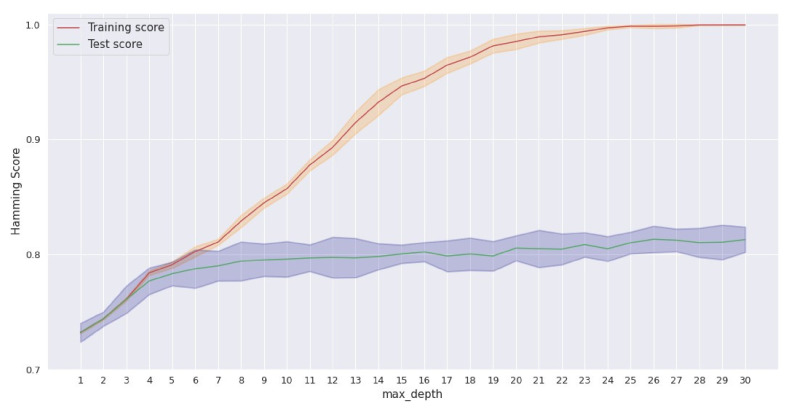
Validation curve for decision tree classifier.

However, in order to find a model with optimal depth in terms of interpretability and performance, a smaller depth of size 4 was chosen, with a small decrease in the score as a trade-off. This choice was based on the results that are depicted in Figure 6. The full representation of the decision tree can be found in the provided notebook (GitHub repository).

### 4.4. Evaluation Metrics

Evaluation of a multi-label classification algorithm is challenging, mostly because multi-label prediction has an additional notion of being partially correct. One trivial way around this would be just to ignore partially correct predictions (consider them as incorrect) and extend the accuracy used in single-label problems for the multi-label case. However, this measure, which is known as the exact match ratio (MR), does not distinguish between completely incorrect and partially correct predictions [28].

Therefore, we used the more appropriate Hamming score or label-based accuracy [29] to evaluate and optimize the models’ performance. The Hamming score is defined as the proportion of the predicted correct labels to the total number (predicted and actual) of labels for that instance. Overall accuracy is the average across all instances. We refer to this metric as accuracy (*A*) or label-based accuracy (LBA) and we compute it using the following formula:

(1) $$Accuracy,\;A=\frac{1}{n}\overset{n}{\underset{i=1}{\sum }}\frac{\left|y_{i}\cap \hat{y_{i}}\right|}{\left|y_{i}\cup \hat{y_{i}}\right|}$$

where *n* is the number of examples, *y_i_* is the true label for the *i*-th instance and $\hat{y_{i}}$ is the predicted label for the *i*-th instance.

### 4.5. Statistical Significance

To compare the accuracy of multiple classifiers on multiple data, we used the Friedman test. Since its result was statistically significant, we calculated the Nemeyi post hoc test to determine the specific differences. To compare two classifiers on multiple measurements, we calculated the Wilcoxon’s signed rank test, which is the non-parametric alternative to the matched-pair t-test. In both cases, the measurements were the resulting accuracy of each classifier in a 10 × 10-cross-validation (CV) scheme. That is, the test consisted of 10 repetitions of 10-fold cross-validation. Therefore, due to the dependency of the samples, the assumptions of their parametric counterparts were not met. Moreover, we chose the Friedman test because the use of its parametric alternative (i.e., repeated-measures ANOVA) to perform classifier evaluation has been discouraged in previous studies due to its sphericity assumption [30]. Similarly, we used the McNemar’s test to compare our classifier with the chosen baseline on a holdout set. The level of statistical significance was set to 0.05 and an effect size calculation was performed for all the relevant tests.

## 5. Conclusions

A dataset was mined from the website of NORMAN Suspect List Exchange [31] with the objective to model the appropriate instrumental method (GC-HRMS, LC-HRMS or both). In total, 1446 descriptors representing the physicochemical characteristics of the substances were generated for the labeled dataset. A multi-step feature selection methodology led to the selection of the eight most relevant features. The prioritized features were used for model selection. The selected model (decision tree) was fine-tuned by optimizing the hyperparameters through the grid search. The outcome of this end-to-end workflow led to a simple model with accuracy that outperformed the baseline implementation. The models were used and will be used in the future to predict the behavior of new substances. The scripts presented in this study are open-source and can be used as building blocks for suspect screening workflows. The generation of better training datasets and the use of more sophisticated statistical approaches that will aim to improve wide-scope screening results remains a future goal. 

## Figures and Tables

**Table 1:** Performance comparison of feature selection strategies under 10-time 10-fold cross-validation. The number of features for each set is given in parentheses. The best performance across each 10-fold CV is highlighted in bold.

	Initial (1446)	Variance (1074)	Correlation (439)	RF Importance (64)	RFECV (57)	Final (8)
1st 10-Fold	80.06 ± 1.49	80.25 ± 2.17	80.18 ± 1.67	80.34 ± 1.05	80.2 ± 0.89	**80.94 ± 1.07**
2nd 10-Fold	80.67 ± 2.08	80.26 ± 1.87	80.56 ± 2.08	80.2 ± 3.01	80.58 ± 2.7	**81.23 ± 2.04**
3rd 10-Fold	80.81 ± 1.17	80.64 ± 1.83	79.48 ± 0.96	80.54 ± 2.04	80.33 ± 2.34	**81.02 ± 1.86**
4th 10-Fold	**81.19 ± 1.81**	80.14 ± 1.67	79.64 ± 1.51	80.35 ± 1.22	80.12 ± 1.52	80.52 ± 1.7
5th 10-Fold	80.66 ± 1.18	81.17 ± 1.23	79.55 ± 1.33	**81.29 ± 1.2**	81.13 ± 1.28	80.98 ± 1.59
6th 10-Fold	80.41 ± 1.12	80.13 ± 1.22	80.82 ± 1.59	81.15 ± 1.3	80.64 ± 1.05	**81.36 ± 1.46**
7th 10-Fold	**81.42 ± 0.58**	80.92 ± 1.61	80.67 ± 1.73	80.12 ± 1.55	80.85 ± 1.59	81.18 ± 1.19
8th 10-Fold	80.63 ± 1.53	**81.02 ± 1.15**	80.95 ± 0.87	80.58 ± 1.61	80.61 ± 2.34	81.01 ± 1.6
9th 10-Fold	**81.13 ± 1.28**	80.25 ± 1.43	80.43 ± 1.44	79.86 ± 1.11	79.76 ± 1.1	81.04 ± 0.96
10th 10-Fold	80.54 ± 1.08	80.24 ± 1.74	80.52 ± 0.92	80.68 ± 1.29	80.68 ± 1.32	**81.03 ± 1.12**

**Table 2:** The *p*-values for each pairwise comparison using Nemeyi post hoc test. The number of features for each set is given in parentheses. The statistically significant differences (*p* < 0.05) are highlighted in bold.

	Initial (1446)	Variance (1074)	Correlation (439)	RF Importance (64)	RFECV (57)	Final (8)
Initial	1.000	0.658	**0.042**	0.386	0.636	0.458
Variance	0.658	1.000	0.679	0.900	0.900	**0.013**
Correlation	**0.042**	0.679	1.000	0.900	0.701	**0.001**
RF Importance	0.386	0.900	0.900	1.000	0.900	**0.003**
RFECV	0.636	0.900	0.701	0.900	1.000	**0.011**
Final	0.458	**0.013**	**0.001**	**0.003**	**0.011**	1.000

**Table 3:** Classification report of rule-based classifier.

	Precision	Recall	F1-Score	Accuracy
GC class	78.79	85.57	82.04	71.56
LC class	61.87	95.84	75.2	60.99
Micro average	69.71	90.18	78.63	
Macro average	70.33	90.71	78.62	
Weighted average	71.21	90.18	78.97	
Samples average	69.77	90.13	75.34	

**Table 4:** Classification report of decision tree classifier.

	Precision	Recall	F1-Score	Accuracy
GC class	82.25	88.23	85.14	76.61
LC class	76.44	95.59	84.95	79.10
Micro average	79.42	91.53	85.05	
Macro average	79.34	91.91	85.04	
Weighted average	79.64	91.53	85.05	
Samples average	81.86	92.35	84.02	

## Data Availability

The data presented in this study are openly available in Zenodo at https://doi.org/10.5281/zenodo.6123973 and the scripts in GitHub at https://github.com/nalygizakis/LCvsGC.

## Supplementary Materials

The following are available online at https://www.mdpi.com/article/10.3390/metabo12030199/s1, Figure S1: Decision tree for GC-MS and LC-MS amenability. Table S1: Predictions for 65,100 environmentally-relevant compounds.

## References

[1] Diaz-Cruz M.S., Lopez de Alda M.J., Lopez R., Barcelo D. (2003). Determination of estrogens and progestogens by mass spectrometric techniques (GC/MS, LC/MS and LC/MS/MS). J. Mass Spectrom..

[2] Barreca S., Orecchio S., Pace A. (2013). Photochemical sample treatment for extracts clean up in PCB analysis from sediments. Talanta.

[3] Barreca S., Busetto M., Colzani L., Clerici L., Daverio D., Dellavedova P., Balzamo S., Calabretta E., Ubaldi V. (2019). Determination of estrogenic endocrine disruptors in water at sub-ng L−1 levels in compliance with Decision 2015/495/EU using offline-online solid phase extraction concentration coupled with high performance liquid chromatography-tandem mass spectrometry. Microchem. J..

[4] Krauss M., Singer H., Hollender J. (2010). LC-high resolution MS in environmental analysis: From target screening to the identification of unknowns. Anal. Bioanal. Chem..

[5] Bletsou A.A., Jeon J., Hollender J., Archontaki E., Thomaidis N.S. (2015). Targeted and non-targeted liquid chromatography-mass spectrometric workflows for identification of transformation products of emerging pollutants in the aquatic environment. TrAC Trends Anal. Chem..

[6] Vinaixa M., Schymanski E.L., Neumann S., Navarro M., Salek R.M., Yanes O. (2016). Mass spectral databases for LC/MS- and GC/MS-based metabolomics: State of the field and future prospects. TrAC Trends Anal. Chem..

[7] Wang M., Carver J.J., Phelan V.V., Sanchez L.M., Garg N., Peng Y., Nguyen D.D., Watrous J., Kapono C.A., Luzzatto-Knaan T. (2016). Sharing and community curation of mass spectrometry data with Global Natural Products Social Molecular Networking. Nat. Biotechnol..

[8] Chiaia-Hernandez A.C., Schymanski E.L., Kumar P., Singer H.P., Hollender J. (2014). Suspect and nontarget screening approaches to identify organic contaminant records in lake sediments. Anal. Bioanal. Chem..

[9] Creusot N., Casado-Martinez C., Chiaia-Hernandez A., Kiefer K., Ferrari B.J.D., Fu Q., Munz N., Stamm C., Tlili A., Hollender J. (2020). Retrospective screening of high-resolution mass spectrometry archived digital samples can improve environmental risk assessment of emerging contaminants: A case study on antifungal azoles. Environ. Int..

[10] Slobodnik J., Dulio V. (2014). NORMAN Association: A Network Approach to Scientific Collaboration on Emerging Contaminants and their Transformation Products in Europe. Transformation Products of Emerging Contaminants in the Environment.

[11] NORMAN Network (2022). NORMAN Database System. https://www.norman-network.com/nds/.

[12] Dulio V., Koschorreck J., van Bavel B., van den Brink P., Hollender J., Munthe J., Schlabach M., Aalizadeh R., Agerstrand M., Ahrens L. (2020). The NORMAN Association and the European Partnership for Chemicals Risk Assessment (PARC): Let’s cooperate!. Environ. Sci. Eur..

[13] Lowe C.N., Isaacs K.K., McEachran A., Grulke C.M., Sobus J.R., Ulrich E.M., Richard A., Chao A., Wambaugh J., Williams A.J. (2021). Predicting compound amenability with liquid chromatography-mass spectrometry to improve non-targeted analysis. Anal. Bioanal. Chem..

[14] Tomczak M., Tomczak E. (2014). The need to report effect size estimates revisited an overview of some recommended measures of effect size. Trends Sport Sci..

[15] Kerby D.S. (2014). The simple difference formula: An approach to teaching nonparametric correlation. Compr. Psychol..

[16] McGraw K.O., Wong S.P. (1992). A common language effect size statistic. Psychol. Bull..

[17] Japkowicz N., Shah M. (2011). Evaluating Learning Algorithms: A Classification Perspective.

[18] Olivier J., Bell M.L. (2013). Effect sizes for 2 × 2 contingency tables. PLoS ONE.

[19] Aalizadeh R., Alygizakis N., Schymanski E., Slobodnik J., Fischer S., Cirka L., NORMAN Network (2020). S0|SUSDAT| Merged NORMAN Suspect List: SusDat. https://zenodo.org/record/3900203#.YhM9ZOhByUk.

[20] Alygizakis N.A., Oswald P., Thomaidis N.S., Schymanski E.L., Aalizadeh R., Schulze T., Oswaldova M., Slobodnik J. (2019). NORMAN digital sample freezing platform: A European virtual platform to exchange liquid chromatography high resolution-mass spectrometry data and screen suspects in “digitally frozen” environmental samples. TrAC Trends Anal. Chem..

[21] Schymanski E.L., Singer H.P., Slobodnik J., Ipolyi I.M., Oswald P., Krauss M., Schulze T., Haglund P., Letzel T., Grosse S. (2015). Non-target screening with high-resolution mass spectrometry: Critical review using a collaborative trial on water analysis. Anal. Bioanal. Chem..

[22] Gago-Ferrero P., Bletsou A.A., Damalas D.E., Aalizadeh R., Alygizakis N.A., Singer H.P., Hollender J., Thomaidis N.S. (2020). Wide-scope target screening of >2000 emerging contaminants in wastewater samples with UPLC-Q-ToF-HRMS/MS and smart evaluation of its performance through the validation of 195 selected representative analytes. J. Hazard. Mater..

[23] Massei R., Byers H., Beckers L.M., Prothmann J., Brack W., Schulze T., Krauss M. (2018). A sediment extraction and cleanup method for wide-scope multitarget screening by liquid chromatography-high-resolution mass spectrometry. Anal. Bioanal. Chem..

[24] Yap C.W. (2011). PaDEL-descriptor: An open source software to calculate molecular descriptors and fingerprints. J. Comput. Chem..

[25] Shi C., Borchardt T.B. (2017). JRgui: A Python Program of Joback and Reid Method. ACS Omega.

[26] USEPA (2022). Mpbpnt.exe Included in Ecological Structure Activity Relationships. https://www.epa.gov/tsca-screeningtools/ecological-structure-activity-relationships-ecosar-predictive-model.

[27] Lehman A. (2005). Jmp for basic univariate and multivariate statistics: A step-by-step guide. Math. Stat. Multivar. Anal..

[28] Sorower M.S. (2010). A literature survey on algorithms for multi-label learning. Comput. Sci..

[29] Godbole S., Sarawagi S. Discriminative methods for multi-labeled classification. Proceedings of the Pacific-Asia Conference on Knowledge Discovery and Data Mining.

[30] Demsar J. (2006). Statistical comparisons of classifiers over multiple data sets. J. Mach. Learn. Res..

[31] NORMAN Network (2022). NORMAN Suspect List Exchange (SLE). https://www.norman-network.com/nds/SLE/.

